# Burden, timing and causes of maternal and neonatal deaths and stillbirths in sub–Saharan Africa and South Asia: protocol for a prospective cohort study

**DOI:** 10.7189/jogh.06.020602

**Published:** 2016-12

**Authors:** AMANHI study group. Maternal, neonatal deaths and stillbirths mortality cohort study

**Affiliations:** AMANHI–Alliance for Maternal and Newborn Health Improvement

## Abstract

**Objectives:**

The AMANHI mortality study aims to use harmonized methods, across eleven sites in eight countries in South Asia and sub–Saharan Africa, to estimate the burden, timing and causes of maternal, fetal and neonatal deaths. It will generate data to help advance the science of cause of death (COD) assignment in developing country settings.

**Methods:**

This population–based, cohort study is being conducted in the eleven sites where approximately 2 million women of reproductive age are under surveillance to identify and follow–up pregnancies through to six weeks postpartum. All sites are implementing uniform protocols. Verbal autopsies (VAs) are conducted for deaths of pregnant women, newborns or stillbirths to confirm deaths, ascertain timing and collect data on the circumstances around the death to help assign causes. Physicians from the sites are selected and trained to use International Classification of Diseases (ICD) principles to assign CODs from a limited list of programmatically–relevant causes. Where the cause cannot be determined from the VA, physicians assign that option. Every physician who is trained to assign causes of deaths from any of the study countries is tested and accredited before they start COD assignment in AMANHI.

**Importance of the AMANHI mortality study:**

It is one of the first to generate improved estimates of burden, timing and causes of maternal, fetal and neonatal deaths from empirical data systematically collected in a large prospective cohort of women of reproductive age. AMANHI makes a substantial contribution to global knowledge to inform policies, interventions and investment decisions to reduce these deaths.

The past two decades have seen significant global declines in both maternal and child mortalities. These declines may be attributable, in part, to the attention they received due to the millennium development goals (MDGs) [1]. However, gains have not always been realized in areas with the highest burden. Three million neonatal deaths, 2.6 million stillbirths and over 280 000 maternal deaths still occur annually [2]. The vast majority (99%) of these deaths continue to occur in low– and middle–income country settings (LMICs); a disproportionate 85% occur in South Asia and sub–Saharan Africa alone [3,4].

An incomplete understanding of the burden, timing and causes of maternal, fetal (stillbirths) and neonatal deaths remains a major challenge in addressing mortality in mothers and their newborns. In many LMICs, routine vital registration systems are lacking or incomplete. Facility and community data are often not systematically collected and medical death certification systems are non–functional. For example, in sub–Saharan Africa and South Asia, less than 12% of countries meet criteria for reasonably complete cause of death reporting [5].

Given these limitations, methods to directly estimate mortality have been developed [6]. These estimates rely on national surveys, demographic surveillance systems or samples of populations to determine causes of deaths [6–10]. While they provide a basis for action, these estimates are subject to biases and limitations of the data sources and may over– or under–estimate the true burden of these deaths [11,12]. Sadly, these are the only sources of evidence to inform planning, prioritization and distribution of resources in many LMICs [12].

## OBJECTIVES

The Alliance for Maternal and Newborn Health Improvement (AMANHI) mortality study aims to determine the burden, timing and causes of maternal deaths, stillbirths and neonatal deaths using harmonized methods across eleven sites in sub–Saharan Africa and South Asia. This prospective study, centrally coordinated by the department of Maternal, Newborn Child and Adolescent Health of the World Health Organization (WHO/MCA), uses harmonized tools, training and implementation strategies across participating sites to collect data from a large cohort of women of reproductive age, their pregnancies and the outcome of these pregnancies for both mothers (up to 42 days postpartum) and their newborns (till end of the neonatal period). The study will also assess the comparability of the estimates generated from these prospectively collected data with existing model–based estimates of maternal mortality, stillbirths and neonatal mortality. The data will also be used to test other innovative approaches (including computer–based “machine learning” methods) to estimate the causes of these deaths. This manuscript describes the protocol for the AMANHI mortality study highlighting key steps that have been implemented to ensure reliability and external validity of the findings.

## METHODS

AMANHI is a multi–centre, multi–country, population–based, cohort study in which women of reproductive age are followed through pregnancy, childbirth and the postnatal period. The AMANHI mortality study includes sites from Bangladesh (Sylhet), India (Haryana and Uttar Pradesh), Pakistan (Karachi and Matiari) in South Asia; and Democratic Republic of Congo (Equator), Ghana (Kintampo), Kenya (Western province), Tanzania (Ifakara and Pemba) and Zambia (South Zambia) in sub–Saharan Africa. A summary description of the sites’ characteristics is provided in **Table 1**. All the sites involved in the AMANHI mortality study were those that had planned or on–going studies on neonatal health funded by the Bill and Melinda Gates Foundation. All these studies planned to enrol greater than 5000 pregnant women, and had established a surveillance system for identifying all pregnant women in a geographically defined area. They also planned to follow up pregnant women through pregnancy and up to 72 hours after birth.

**Table 1:** Summary description of the parent studies, surveillance system, surveillance population and annual number of births at AMANHI sites

Site	Parent study title and objective	Existing pregnancy surveillance system	Total surveillance population	Reproductive–aged women in surveillance	Approximate annual births
Bangladesh	Aetiology of Neonatal Infection in South Asia (ANISA): to estimate community level aetiology–specific incidence predictive risk factors and clinical features, treatment and prevention strategies for serious infections among young infants (0–59 d).	2–monthly by trained community health workers (CHWs)	600 000	88 000	13 000
Democratic Republic of Congo (DRC)	African Neonatal Sepsis Trial (AFRINEST): to test the safety and efficacy of simplified antibiotic regimens for treating possible serious bacterial infection in 0–59 day–old infants.	3–monthly by CHWs	699 288	65 000	12 000
Ghana	Neonatal vitamin A supplementation (NeovitA) study: to determine if vitamin A supplementation to neonates once, orally, <48 hours of birth will reduce neonatal, early and late infant mortality.	Monthly by fieldworkers	700 000	147 000	21 000
India – Haryana	NeovitA study: same as Ghana.	Monthly by trained CHWs	1 400 000	313 399	34 600
India – Shivgarh	Topical emollient application to babies to prevent infection especially in pre–terms & ANISA studies.	3–monthly by fieldworkers	1 350 000	184 430	44 000
Kenya	AFRINEST study: same as DRC.	3–monthly by CHWs	400 000	30 000	10 000
Pakistan – Karachi	ANISA study: same as Bangladesh.	3–monthly by fieldworkers	270 000	63 000	9500
Pakistan–Matiari	ANISA study: same as Bangladesh.	3–monthly by fieldworkers	215 200	64 000	8000
Tanzania–Ifakara	NeovitA Study: same as Ghana.	3–monthly by fieldworkers	300 000	72 000	6000
Tanzania–Pemba	Chlorhexidine (CHX) study: to evaluate the efficacy of chlorhexidine cord cleansing on neonatal mortality.	6 weekly by trained CHWs	390 000	72 000	14 000
Zambia*	Chlorhexidine (CHX) study: to evaluate the efficacy of chlorhexidine cord cleansing on neonatal mortality.	No pregnancy surveillance; facility ANC enrolment	25 000	25 000	9000

*Zambia to recruit only from antenatal clinics.

### Training for harmonized implementation

The AMANHI mortality study teams undertook two main training sessions, facilitated by experts from the WHO, in Geneva Switzerland to harmonize the implementation of study procedures. The first session in June 2012 involved principal investigators from sites. At this training workshop, sites developed common data collection tools (core variable tables) and implementation strategy. Sites adapted the generic protocol to suit their context and submitted to the Ethics review committees of the WHO/MCA and other relevant institutions.

In August 2014, AMANHI brought together two site coordinators per site for a week–long training to harmonize physician assignment of causes of deaths (CODs). Participants used principles of the International Statistical Classification of Diseases (ICD) to assign CODs and complete death certificates. Participants used these principles contained in an AMANHI VA manual to practice until they assigned the same CODs for five consecutive forms. These participants, in turn, trained physicians in their respective sites.

### Mortality surveillance

The AMANHI mortality surveillance utilizes an existing 1–6 monthly routine household surveillance visits by trained fieldworkers to over 2 million women of reproductive age across sites to identify and follow–up pregnant women through pregnancy, childbirth to 42 days postpartum. The surveillance comprises active and passive components. In the former, fieldworkers identify pregnant women, obtain their consent and enrol them for follow–up, providing them with unique study identification numbers (study ID). The study will therefore obtain data on all women who become pregnant, every pregnancy and their outcomes (including abortions/miscarriages, stillbirths and livebirths). These will serve as denominators for estimating rates of maternal, fetal and neonatal mortality. Fieldworkers record all maternal, fetal/stillbirth or neonatal deaths that occur among enrolled participants during surveillance visits. A listing of these deaths is used by specially trained supervisors for the VA interviews. This list will be used to generate numerators for the mortality rate estimates after the type (maternal, pregnancy–related, fetal or neonatal) and timing (details below) are confirmed from the VA interviews. Fieldworkers also collect baseline socio–demographic data and assets inventory for classifying households into wealth quintiles. This will allow for evaluation of inequities in the distribution of mortality burden within the population.

The primary study outcomes include all–cause maternal mortality ratio (MMR) defined as number of women who die whilst pregnant or within 42 days of a pregnancy’s end, irrespective of the duration or site of the pregnancy per 100 000 livebirths. Stillbirth rate (SBR) will also be calculated either as the number of stillbirths per 1000 births (true rate) or per 1000 livebirths (ratio). Neonatal mortality rate (NMR) will be defined as the number of deaths among live born infants within the first 28 days after birth per 1000 live births. Timing of maternal deaths will be classified as deaths in early pregnancy, late pregnancy, intrapartum, immediate postpartum and late postpartum; stillbirths will be classified as antenatal or intrapartum and neonatal deaths by day of death for each day in the first week after birth and then weekly till day 28. Cause–specific mortality rates/fractions will also be determined. The data will also be disaggregated and rates estimated separately for sub–Saharan Africa and South Asia.

In nine of the eleven sites where a maternal morbidity surveillance runs concurrently, women receive five scheduled visits–three in pregnancy and two postpartum. Mortality surveillance is incorporated into these visits. The fieldworkers review health facility records to identify mortality events for VA interviews. In the interval between visits, families report deaths or pregnancy losses to AMANHI for follow–up (passive surveillance). The Zambia site is the only exception because pregnancy identification is only done at antenatal clinics. This approach was used because of high antenatal care coverage (over 96%) within the study district [13].

### The AMANHI Verbal Autopsies: confirming type, timing and obtaining causes of deaths

In all sites, when a stillbirth occurs, a baby or woman of reproductive age dies, trained VA supervisors visit the household, after the mourning period, and conduct VA interviews to obtain detailed information on the circumstances leading to the death. The VA supervisors identify a reliable informant, defined as any person who lived closely with the deceased in the period immediately preceding the death and who is capable of providing reliable and coherent account of the circumstances leading to the death, for the interview. The objectives for administering the AMANHI VAs are three–fold: first, to confirm deaths and the type of death especially the critical discrimination between maternal or pregnancy–related deaths (for women) and between stillbirths and early neonatal deaths. Second, the VAs will also confirm the timing of the deaths as described in the previous section. The third objective is to assign causes to the deaths. AMANHI uses a uniform tool and harmonised methods for the collection and interpretation of the VA data.

### The AMANHI VA tool

Principal investigators from each site, together with the WHO/MCA coordinating team, developed a table of core variables to be collected across sites for all deaths. These variables were derived from three existing tools: the WHO VA [14], InterVA [15] and SMARTVA (Tariff method) [16] tools. The WHO tool was used as the template and questions from the other tools were added if they were not already in this template. When questions were found to be similar but response options differed between tools, both questions were maintained in the AMANHI tool. This will allow for data generated in AMANHI to be analysable using these top two available software platforms (InterVA and SMARTVA). The AMANHI core variable table therefore includes questions to be asked, the response options and how variables should be captured in the final common study database. This harmonised data collection tool will also facilitate pooling of data across sites and hence increase statistical power for analysis on rarer outcomes.

### AMANHI VA interviews–form completion

Questions in core variable tables were translated into site–specific questionnaires in three sections: a narrative, close–ended questions and records review and data abstraction sections.

**Semi–structured narratives.** Interviewers ask respondents to provide detailed, chronological narratives on the circumstances leading to the death. Where needed, they further probe for specific details about current or any pregnancies that had ended around the time of death; the onset of any illness, signs and symptoms exhibited, any pre–existing medical conditions and care–seeking during the pregnancy and/or fatal illness. Irrespective of pregnancy outcomes, interviewers probe into pregnancy and labour history, whether a baby was stillborn or died after birth. Where technical or local words are used for signs they probe and write down what the respondent meant rather than their own interpretations.

**Close–ended questions.** Interviewers collect basic demographic and socio–economic characteristics of the deceased and systemically elicit responses for all signs and symptoms that the deceased exhibited before death. These close–ended questions as well as providing details on some of the signs and symptoms also elicit important signs and symptoms that respondents may not mention in the narratives. For instance, for haemorrhage, they probe for the onset, severity, duration and any care sought and the outcomes of the care–seeking. In case the narrative conflicts with the close–ended responses, interviewers probe further to ensure data are internally consistent.

**Records review and abstraction.** Interviewers abstract relevant data from hospital, antenatal, childbirth, postpartum clinic attendance records or death certificates onto the VA questionnaire.

### Interpreting AMANHI VAs through harmonised assignment of causes of deaths

The AMANHI mortality study uses harmonized protocols (in an AMANHI VA manual) to assign CODs. This is to improve objectivity and transparency of the COD assignment and increase validity and reliability of physician–assigned causes. The manual provides uniform criteria and processes for selection and training of physicians; common definitions and procedures for assigning causes [17]; centralised accreditation and certification of trained physicians and streamlining the entire process on a specially–designed software platform.

**Training and accreditation of physicians.** PIs and study coordinators recruited and trained selected local physicians on the principles of AMANHI VA using the VA manual. A list of all trained physicians is then submitted to the WHO/MCA for accreditation. The trained physicians were provided online access to 20 standardised VA forms (stillbirths/neonatal–12 and women of reproductive age–8) that had CODs assigned by global VA experts. The numbers were so selected to reflect the relative frequency of occurrence of these deaths as well as provide enough numbers to test a variety of cases. Upon completion, the physicians submit the forms online to the WHO/MCA who compare the physician assigned CODs with the standard CODs for agreement. Physicians are only accredited when 80% or more of their assigned CODs agree with the standard. The 80% mark was selected because we considered that one in every five forms may be difficult to code due to poor quality of data. Physicians are given three attempts at accreditation and when they fail, they are not allowed to assign CODs in AMANHI. After each unsuccessful attempts, coordinators and an expert from WHO retrained physicians. AMANHI certificates were given to all accredited physicians.

**Assigning CODs.** The study employed ICD principles adapted from the revised WHO Verbal Autopsy Coding Standards (2012) [14,17]. A list of programmatically–relevant causes of maternal, fetal and neonatal deaths (**Table 2****,** **Table 3** and **Table 4**) were selected and their operational definitions for AMANHI were specified. When the cause is known but not included in the AMANHI list, an option is given to code as such or as indeterminate if no COD can be assigned.

**Table 2:** AMANHI list of underlying causes of pregnancy–related deaths

Underlying cause of pregnancy–related deaths:
Ectopic pregnancy
Abortion–related death
Pregnancy–induced hypertension (pre–eclampsia)
Pregnancy–induced hypertension (eclampsia)
Obstetric haemorrhage (antepartum)
Obstetric haemorrhage (postpartum)
Obstructed labour
Ruptured uterus
Pregnancy–related sepsis (antepartum)
Pregnancy–related sepsis (postpartum)
Severe anaemia
Pre–existing medical conditions exacerbated by pregnancy
Accidents/injuries
Other specific obstetric causes
Other specific NON–OBSTETRIC causes
Cause not possible to determine from verbal autopsy
**Other significant conditions contributing to death:**
Anaemia
Severe malnutrition
HIV
Maternal age <15 years
Maternal age >35 years

**Table 3:** AMANHI list of underlying causes of neonatal deaths and contributing conditions

Underlying cause of neonatal death:
Preterm birth complications
Perinatal asphyxia
Neonatal pneumonia
Neonatal sepsis/meningitis
Neonatal tetanus
Congenital malformations
Neonatal diarrhoea
Accidents/injuries
Other specific perinatal causes
Cause not possible to determine from verbal autopsy
**Other conditions contributing to neonatal death*:***
Term low birthweight (small for gestational age)
Prematurity
**Maternal condition leading to neonatal death:**
Pregnancy–induced hypertension (pre–eclampsia)
Pregnancy–induced hypertension (eclampsia)
Obstetric haemorrhage (antepartum)
Obstructed labour
Ruptured uterus
Maternal infection affecting the baby
Pre–existing medical conditions exacerbated by pregnancy
Accidents/injuries
Other obstetric complications (malpresentation, cord prolapse)
Other specific maternal conditions
No identifiable maternal conditions

**Table 4:** AMANHI list of types and underlying causes of fetal deaths (stillbirths) and contributing conditions

Maternal/foetal underlying condition:
Congenital malformations
Pregnancy–induced hypertension
Gestational diabetes
Antepartum haemorrhage
Maternal infections that can affect the foetus
Maternal medical conditions (diabetes, epilepsy, etc.)
Maternal accident/injury
Obstructed labour
Other obstetric complications (malpresentation, cord prolapse)
Other specific perinatal causes
Cause not possible to determine from verbal autopsy
**Other conditions contributing to the stillbirth:**
Small–for–date baby
Multiple pregnancy
Post–date (>10 months)
Maternal age <15 years
Maternal age >35 years
Obesity
Severe malnutrition
Smoking, alcohol or drug abuse

**Figure 1:**
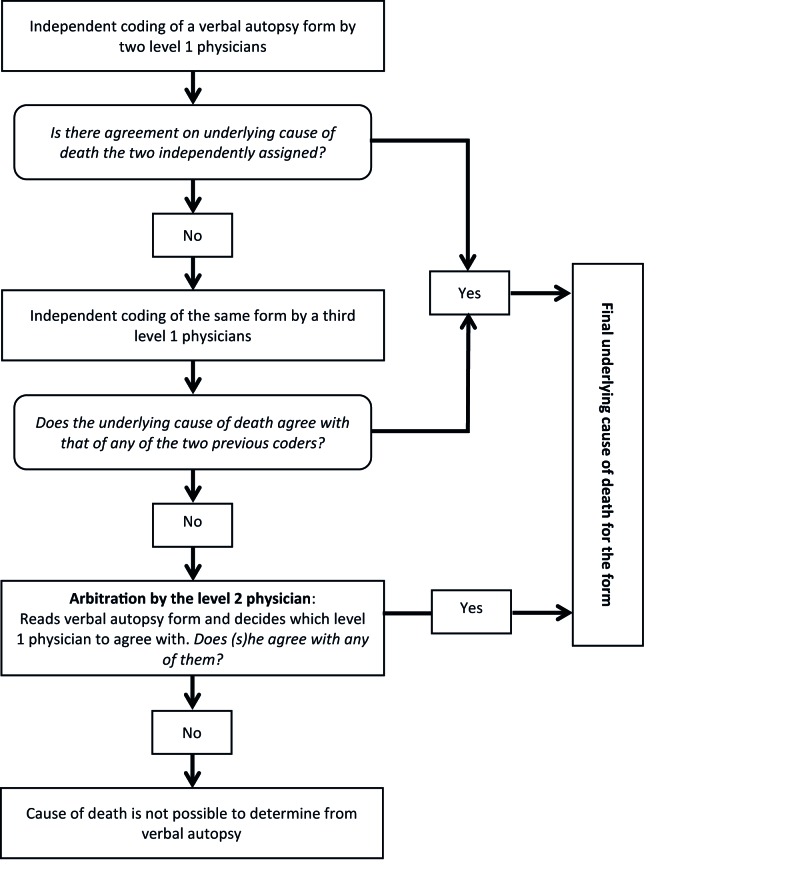
Algorithm for consensus building around cause of death in AMANHI.

**Procedure for consensus building.** The AMANHI algorithm for the process of consensus building around CODs is shown in **Figure 1**. The underlying cause of death (UCOD) assigned by physicians is used for consensus building. In AMANHI, at least two out of four physicians must agree on a cause to be assigned as final UCOD. Physicians are classified at two levels based on clinical and previous VA coding experience. Two level 1 physicians (practitioners who routinely manage pregnant women and newborns) first code each VA form independently followed by a third level 1 physician if their assigned UCODs differ. When all three level 1 physicians do not agree, the form is elevated to a level 2 physician (specialists in obstetrics, neonatal or child health and/or very experienced in VA coding) for arbitration. If the level 2 physician agrees with the UCOD assigned by any of the level 1 physicians, that cause is assigned to the death. However, when they do not agree with all three, the form is coded as indeterminate. The level 2 physicians also determine whether it was a neonatal or fetal death and, for the latter, whether it was ante– or intrapartum. All physicians also assign immediate and antecedent causes of deaths for each VA death certificate and specify co–existing significant pathologies/conditions that might have contributed to the death. They draw a flow diagram to explain the link between UCOD and the antecedent and immediate cause(s).

**AMANHI verbal autopsy software and quality control of the coding process.** A customized software platform was developed by the Community Empowerment Laboratory (Lucknow, Uttar Pradesh, India), in collaboration with WHO/MCA to facilitate the COD assignment. The software helps to coordinate and manage the coding process. It has in–built algorithms to automate the assignment of forms to physicians and for the consensus–building process (**Figure 1**). Its user interface groups clinical signs and symptoms on the VA form according to physiological systems or/and stages of pregnancy. It also provides physicians with the template to construct the flowchart on the mechanism of the death and mandates them to complete a death certificate for each death, providing the list of UCODs in a dropdown menu. As a monitoring tool, site coordinators have a visual display of the frequency of agreement between each physician and the final UCOD for every form they code and this is used as proxy index to guide refresher training needs of physicians.

### Quality monitoring

AMANHI–specific quality control procedures include physical presence of study supervisors to directly observe 5% VA interviews as they are being conducted in the field. They then provide prompt feedback on fieldworker performance. Also, immediately after collection on the field, data are manually checked for completeness and consistency before transmission for data entry. The WHO/MCA sends experts on 6–monthly site visits to monitor quality of implementation. AMANHI mandates every site to submit monthly progress report and transmit all collected data every quarter for quality review and feedback.

### Sample size considerations

Approximately 263 000 pregnant women will be enrolled into the mortality study across the 11 sites: about 126 000 from sub–Saharan Africa and 137 000 from South Asia. Sample size considerations were based on maternal mortality, given the rarity of this outcome. Estimated regional MMRs for sub–Saharan Africa and south Asia, pooled from the included countries, were 435 and 290 per 100 000 livebirths respectively (**Table 5**). With these sample sizes, AMANHI would have more than 90% power, at the 5% significance level, to detect all–cause mortality with a precision of ±8% for sub–Saharan Africa and ±10% for south Asia, with a higher precision for the pooled sample across all sites. The study will also have adequate power to quantify any single cause that accounts for at least 20% mortality (within ±15%). Considerations for country–specific samples sizes are shown in **Table 5**. With relatively more common outcomes such as stillbirths and neonatal deaths these sample sizes will guarantee highly precise mortality rate estimates overall and for regions and countries.

**Table 5:** Site specific sample size for all cause maternal mortality

Study site	Sample size	Estimated maternal mortality ratio/100 000 live births	Relative precision
**Sub region–Sub–Saharan Africa:**			
Democratic Republic of the Congo	20 000	670	±17%
Ghana	32 000	250	±22%
Kenya	22 000	400	±21%
Tanzania (2 sites)	27 000	360	±20%
Zambia	25 000	590	±16%
**Sub–Saharan Africa (pooled)**	**126 000**	**435**	**±8%**
**Sub–region–South Asia:**			
Bangladesh	19 000	200	±32%
India (2 sites)	90 000	325	±11%
Pakistan (2 sites)	28 000	240	±22%
**South Asia (pooled)**	**137 000**	**290**	**±10%**

### Data management

Data are collected using paper–based forms or tablet–based software with the exception of Zambia where field monitors collected data using forms designed in the TeleForms^®^ system (HP, Cambridge, UK). After supervisors in Zambia review forms for completeness, they scan them, enter and export all the data into an Access database for management. Narratives are transcribed in the language of collection or directly into English, French, Swahili, Hindi or Urdu. Close–ended questions are double–entered independently by two clerks into appropriate software with in–built range and consistency checks. The double–entry checks against entry errors. All data are saved to a dedicated password–protected server and transferred quarterly to the WHO/MCA for further consistency checks.

### Data analysis

All analyses will be conducted using Stata statistical software [18]. Simple tabulations will be done to describe the overall burden, timing and causes of deaths–maternal, stillbirth and neonatal. Estimates will also be generated from the sub–sample of women who were also part of the prospective morbidity follow–up.

### Ethical clearance and informed consent

The AMANHI mortality study received ethical clearance from institutional review boards in the participating countries, host institutions of principal investigators (including Johns Hopkins University, University of Kinshasa, London School of Hygiene and Tropical Medicine and Boston University) and the WHO. Informed consent is obtained from all respondents to the VA interviews.

## IMPORTANCE OF THE AMANHI MORTALITY STUDY

The AMANHI Mortality study is one of the first to generate improved estimates of the burden and timing of maternal, fetal and neonatal deaths in sub–Saharan Africa and south Asia from empirical data systematically collected in a large prospective cohort of women of reproductive age. More critically, it will make substantial contributions to global knowledge on the causes of these deaths. These improved estimates will inform policy, interventions and investment decisions to reduce these deaths.

Availability of robust data are critical to intervention design and implementation. In resource–limited settings, allocation of scarce resources requires evidence–based decision–making which must be informed by reliable data [4,19]. Current evidence is derived from estimates from statistical models based largely on cross–sectional data, often facility based and generated using different methodologies and with varying definitions of outcomes. As a result of biases in the data, estimates from these models are difficult to validate, limiting stakeholder buy–in and adversely affecting their use in planning, intervention design and policy decision–making [4,12,20].

The AMANHI mortality study has many strengths. It is a population–based prospective study which followed up a large cohort of women of reproductive age at the community level. Data were also collected from the facilities women attended for deliveries and medical emergencies. This will therefore reduce selection biases that might have resulted from using only facility–based data. Perhaps the greatest strength is the harmonised implementation across all 11 study sites. With the use of common tools and definitions of variables and outcomes, AMANHI collected context–relevant but uniform data across all sites. This validates the pooling of the data across the sites to allow for analyses on rare outcomes.

The AMANHI approach to assigning causes of deaths strategically addresses many of the drawbacks in the use of physicians including drain on their time and subjectivity. The AMANHI VA coding software reduced physician coding time from 90 minutes to less than 20 minutes per verbal autopsy. This reduced time addresses concerns by some advocates that physician times should be better spent in actual service delivery [15,21–23]. Besides, whilst automated computer algorithms have the potential to dramatically improve the speed and efficiency of classifying causes and reducing cost [24], incorporating physician’s knowledge of the local context, terminologies and their interpretations in evaluating the causes of deaths is an additional advantage that computer–based algorithms may not have. Furthermore, the use of the ICD principles, centralised accreditation process, automation of the form assignment to physicians and other quality control mechanisms improved the transparency and objectivity of the process. The provision of a limited list of causes and their operational definitions also reduced the occurrence of false discordance between physicians due to typographical errors.

One major limitation of the AMANHI mortality study is generalizability of the results. Data were collected in defined parts of the respective countries; at sites systematically selected for newborn health interventions because of high mortality rates. It is plausible that mortality rates in the study areas might not truly represent all regions of the participating countries. However, estimates from AMANHI will be compared to prevailing estimates for countries. Differences may result from several factors such as quality of data but will challenge the status quo.

The need for robust empirically generated data to inform policy and planning in LMICs is long overdue. By generating data from a very large cohort of women across eleven countries in these two geographic areas with the highest mortality burden, AMANHI will provide very precise estimates of mortality, their timing and causes to inform researchers and policy–makers on improved methods for assigning cause of death in women and children.
